# Pharmacodynamics of evocalcet for secondary hyperparathyroidism in Japanese hemodialysis patients

**DOI:** 10.1007/s10157-018-1635-6

**Published:** 2018-08-29

**Authors:** Takashi Shigematsu, Ryutaro Shimazaki, Masafumi Fukagawa, Tadao Akizawa, Evocalcet Study Group

**Affiliations:** 1 Department of Nephrology, Wakayama Medical University, 811-1 Kimiidera, Wakayama City, Wakayama 641-8509 Japan; 2 R&D Division, Kyowa Hakko Kirin Co., Ltd., 1-9-2 Otemachi, Chiyoda-ku, Tokyo, 100-0004 Japan; 3 Division of Nephrology, Endocrinology and Metabolism, Department of Internal Medicine, Tokai University School of Medicine, 143 Shimokasuya, Isehara-shi, Kanagawa, 259-1193 Japan; 4 Division of Nephrology, Department of Medicine, Showa University School of Medicine, Namics 301, 4-24-51 Takanawa, Minato-ku, Tokyo, 108-0074 Japan

**Keywords:** Calcimimetics, Evocalcet, Hemodialysis, Parathyroid hormone, Pharmacodynamics, Secondary hyperparathyroidism

## Abstract

**Background:**

This study investigated the pharmacokinetics, pharmacodynamics, and safety of multiple doses of evocalcet in Japanese secondary hyperparathyroidism (SHPT) patients receiving hemodialysis.

**Methods:**

In this multicenter, open-label study, conducted between August 2013 and March 2014, 27 patients received multiple doses of 1 and 4 mg evocalcet for 14 days, followed by an extension period of multiple doses of 8 and 12 mg evocalcet for 7 days using an intra-patient dose escalation protocol. Pharmacodynamic parameters consisted of measurement of intact parathyroid hormone (PTH), serum-corrected calcium, serum phosphorus and intact fibroblast growth factor 23 concentrations. Safety was assessed by analysis of adverse events.

**Results:**

Plasma evocalcet levels reached steady state 3 days after the first day of administration. Pharmacodynamic analyses showed that evocalcet effectively reduced intact PTH and serum-corrected calcium levels. Adverse events (AEs) occurred in 29.6 and 62.5% of patients receiving multiple doses of 1 or 4 mg, respectively. The AE ‘blood calcium decreased’ occurred in eight patients (33.0%) after multiple doses of 4 mg. All events were mild, except for one patient with a moderate AE (abnormal liver function) and one patient with a severe adverse drug reaction (blood calcium decreased).

**Conclusion:**

Multiple doses of evocalcet reduced intact PTH levels with a concomitant decrease in serum calcium levels. Evocalcet was well tolerated in SHPT patients receiving hemodialysis.

**Electronic supplementary material:**

The online version of this article (10.1007/s10157-018-1635-6) contains supplementary material, which is available to authorized users.

## Introduction

Secondary hyperparathyroidism (SHPT) is a complication of chronic kidney disease (CKD) in patients undergoing hemodialysis, caused by the parathyroid gland overproducing parathyroid hormone (PTH) [1, 2]. Excess levels of PTH are associated with chronic kidney disease–mineral and bone disorder (CKD–MBD), which results in a loss of calcium from the bones with the subsequent increase of calcium levels in the blood [3–5]. This in turn causes systemic toxicity and is associated with arterial calcification and cardiovascular diseases such as atherosclerosis [6–8].

The Japanese Society for Dialysis Therapy guidelines for the management of CKD–MBD recommend the maintenance of serum phosphorus, serum calcium, and serum PTH concentrations within specified ranges. The calcimimetic cinacalcet hydrochloride is a positive allosteric modulator of the calcium-sensing receptor that suppresses PTH secretion [9, 10]. At present, cinacalcet hydrochloride is the preferred therapeutic option for lowering PTH levels while maintaining normal serum calcium and phosphorus concentrations, and improving mortality and cardiovascular outcomes in CKD patients [9, 10]. However, cinacalcet hydrochloride is commonly associated with gastrointestinal (GI) adverse events (AEs), such as nausea and vomiting, which have been reported to cause poor drug compliance, resulting in inadequate therapeutic levels of treatment [11, 12].

Evocalcet is expected to be a novel, oral, positive allosteric modulator of the calcium-sensing receptor for the treatment of SHPT patients undergoing hemodialysis. A pre-clinical study showed that evocalcet had a favorable profile of suppressing PTH secretion with GI AEs [13]. Recently, a phase I clinical study showed that no GI-related AEs occurred in healthy Japanese male adults after single and multiple doses of evocalcet (manuscript in preparation). Therefore, the present study aimed to examine the pharmacodynamics and safety of evocalcet administered orally in multiple doses in SHPT patients receiving hemodialysis. This study was initiated with single doses of evocalcet, which is described elsewhere [14]. Using an intra-patient escalation method, SHPT patients were then transitioned to a 14-day multiple-dose period followed by a 7-day extension period.

## Methods

### Patients

SHPT patients receiving hemodialysis entered the multiple-dose and extension periods after receiving evocalcet at least once during the single-dose period [14]. The inclusion criteria were age ≥ 20 and < 75 years at the time of consent, hemodialysis three times per week for ≥ 12 weeks prior to screening, iPTH level of ≥ 240 pg/mL, and serum calcium levels corrected for albumin ≥ 8.4 mg/dL at screening. Cinacalcet was washed out ≥ 2 weeks before screening. The dose and mode of administration of active vitamin D, phosphate binders, or calcium preparations, and the dialysis condition (dialysate calcium level, blood purifier, prescribed duration of dialysis, prescribed frequency of dialysis) were not changed ≥ 2 weeks before screening. Exclusion criteria are listed in Supplementary Text S1.

### Study design

**Fig. 1:**
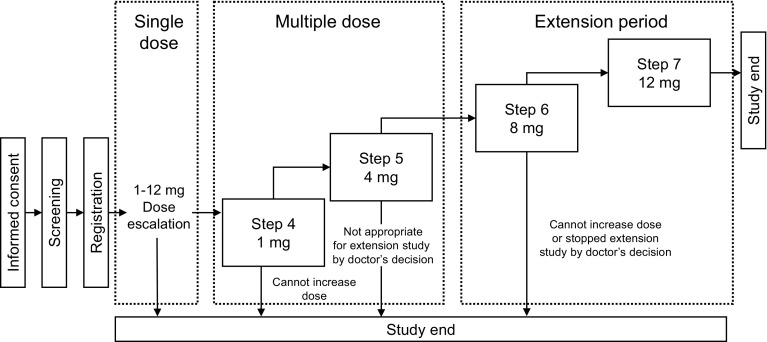
Study design

This was a phase Ib/IIa, multicenter, open-label study evaluating the pharmacodynamics, safety, and pharmacokinetics of multiple doses of evocalcet in Japanese SHPT patients receiving hemodialysis. This study was conducted across 20 centers between August 2013 and March 2014. Figure 1 shows the study design and illustrates how patients transitioned through an intra-patient escalation method from the single-dose period to the multiple-dose period and then finally to the extension period. The escalation criteria for patients to transition from Step 1 through to Step 7 are described in the Supplementary Text S2.

Evocalcet was administered once daily for 14 days during Steps 4 and 5 in the multiple-dose period, and then once daily for 7 days during Steps 6 and 7 in the extension period. During the multiple-dose and extension periods, each step was started on the first dialysis day after the maximum interdialytic interval, and evocalcet was administered before dialysis was initiated on that day. After each step, evocalcet was washed out before commencing the next step.

Use of cinacalcet; changes in the dose or mode of administration of active vitamin D, phosphate binders, or calcium preparations; or changes in the dialysis condition were prohibited from 2 weeks before the screening until the end of the study period.

### Pharmacokinetic analysis

The secondary endpoints included pharmacokinetic evaluation of plasma evocalcet concentration. Blood sampling was carried out on the following days: day 1 (before and 1–3 h after administration of evocalcet and during dialysis); days 3, 5, 8, and 12 (before administration of evocalcet on each day); day 15 (before the start of dialysis and on each scheduled day of visit); and at the time of discontinuation. During the multiple-dose period, from 1 to 3 h after the administration of evocalcet and during dialysis, blood samples were collected simultaneously on the arterial and venous sides of the dialyzer to assess evocalcet clearance. During the extension period, blood sampling was carried out at: days 1, 3, and 5 (before administration of evocalcet on each day); at day 8 (before the start of dialysis); and at the time of discontinuation.

### Pharmacodynamic analysis

The secondary endpoints also included blood pharmacodynamic analysis of intact PTH, whole PTH, ionized calcium, serum-corrected calcium, serum phosphorus, intact fibroblast growth factor 23 (FGF23), and calcitonin. Intact PTH was measured using an ECLusys PTH kit (Roche Diagnostics K. K., Tokyo, Japan) and intact FGF23 was measured using the human FGF23 ELISA kit (KAINOS Laboratories, Inc., Tokyo, Japan).

### Safety analysis

The primary endpoint of this study was to determine the safety profile of evocalcet during the administration of multiple doses in SHPT patients undergoing hemodialysis. This included evaluation of AEs, biochemical determinations (hematology and blood biochemistry), vital signs, 12-lead electrocardiogram (ECG) measurements, and ophthalmologic examinations. All AEs were assessed either by investigators’ interviews or patients’ self-reports with regards to their severity, outcome, and relationship to evocalcet.

Safety profile assessments were carried out on day 1 (before administration of evocalcet and before dialysis on that day) and again on a subsequent examination day (before administration of evocalcet and before dialysis on that day).

### Statistical analysis

The target sample size was 20 to ensure an adequate number of patients to assess the pharmacodynamics, safety, and pharmacokinetics of evocalcet administered repeatedly.

For intact PTH, whole PTH, ionized calcium, serum-corrected calcium, serum phosphorus, intact FGF23, and calcitonin levels, time profiles were prepared at each assay time point during each treatment period by dose. Time profiles were prepared at the end of the final administration and these were also calculated by dose.

In the analysis of pharmacodynamic endpoints at the end of the final administration in each treatment period/dose (step) during the multiple-dose and the extension periods, missing data were imputed using the last observation carried forward measure.

For all AEs which occurred after the start of study treatment, occurrence and incidence of AEs and adverse drug reactions (ADRs) were summarized by each treatment period and dose. Incidence of AEs and ADRs was summarized by MedDRA/J System Organ Classes, preferred term, for each treatment period and dose.

## Results

### Patients

Twenty-nine SHPT patients receiving hemodialysis were initially enrolled into the single-dose period. Two SHPT patients discontinued the study during the single-dose period because of the use of a prohibited concomitant drug (cinacalcet) and an AE. The remaining 27 patients entered the multiple-dose period. The baseline demographics of the enrolled patients are shown in Table 1a. The disposition of patients who transitioned to each step during the multiple-dose and extension periods is summarized in Table 1b.

**Table 1:** Patient baseline demographics and clinical characteristics (a) and disposition of patients at each step (b)

(a)											
Characteristics					(*N* = 29)						
Sex, male					21 (72.4)						
Age, years (mean ± SD)					62.8 ± 9.2						
≥ 65 years					13 (44.8)						
Dry weight, kg (mean ± SD)					58.4 ± 13.1						
Body mass index (kg/m^2^)					22.9 ± 3.2						
Duration of dialysis, months (mean ± SD)					149.4 ± 122.6						
Previous use of cinacalcet											
Cinacalcet hydrochloride					14 (48.3)						
Other calcimimetics					3 (10.3)						
Primary disease											
Diabetic nephropathy					7 (24.1)						
Chronic glomerulonephritis					14 (48.3)						
Nephrosclerosis					2 (6.9)						
Comorbidities											
Diabetes					7 (24.1)						
Type of dialysis											
Hemodialysis					22 (75.9)						
Hemodiafiltration					5 (17.2)						
Other					2 (6.9)						
Intact PTH, pg/mL (mean ± SD)					271.3 ± 109.1						
Corrected calcium, mg/dL (mean ± SD)					9.78 ± 0.50						
Phosphorus, mg/dL (mean ± SD)					4.76 ± 0.79						

(b)											
	Step 4 (1 mg multiple)		Step 5 (4 mg multiple)			Step 6 (8 mg extension)			Step 7 (12 mg extension)		
Consented	35										
Ineligible or dropout	6										
Enrolled	29										
Dropout during the single-dose period	2										
Received study drug	27(93.1%)		24(82.8%)			5(17.2%)			4(13.8%)		
Discontinued the study	1(3.4%)		5(17.2%)			0			0		
Reason											
AE (blood calcium decreased)	0		3			0			0		
AE (other)	1		1			0			0		
Investigator’s decision	0		1			0			0		
Completed the step	26(89.7%)		19(65.5%)			5(17.2%)			4(13.8%)		
Escalation criteria for initiation of the next step											
Not applicable	26		5			4					
Decreased corrected calcium (< 7.5 mg/dL)	0		5			0					
Investigator’s decision	0		9			1					

*Two of these patients conflicted with the escalation criteria during the single-dose period and, therefore, did not proceed to Step 5

### Pharmacokinetic analysis

**Fig. 2:**
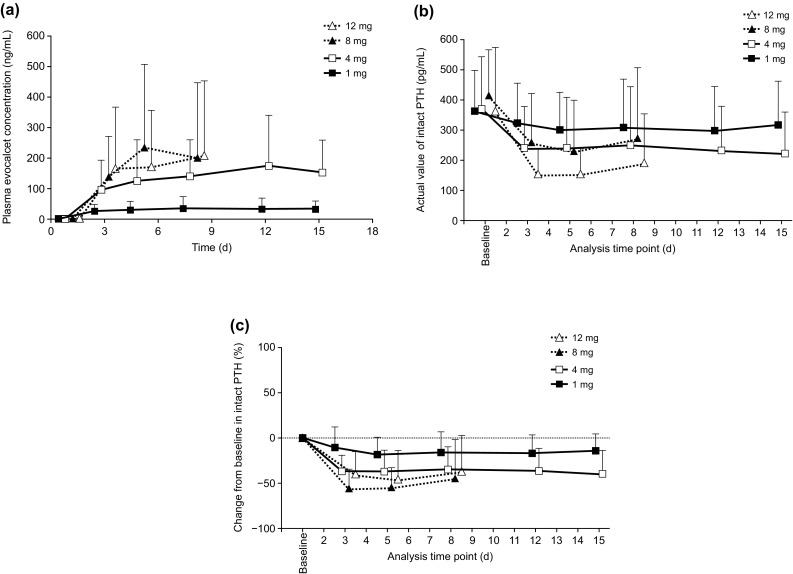
Change in plasma evocalcet concentration (*C*_trough_) (**a**), and the actual value of (**b**) and the percent change in (**c**) intact PTH levels over time after multiple doses. *PTH* parathyroid hormone

The change in plasma evocalcet concentration, as measured by *C*_trough_ over time, was almost constant at any dose from day 3 to the end of the treatment (Fig. 2a). There were also no marked differences in plasma evocalcet concentration levels between males and females.

Plasma samples collected from the arterial and venous sides of the dialyzer showed that evocalcet concentrations were similar, suggesting that even with multiple doses evocalcet was not being cleared by hemodialysis (data not shown).

### Pharmacodynamic analysis

**Fig. 3:**
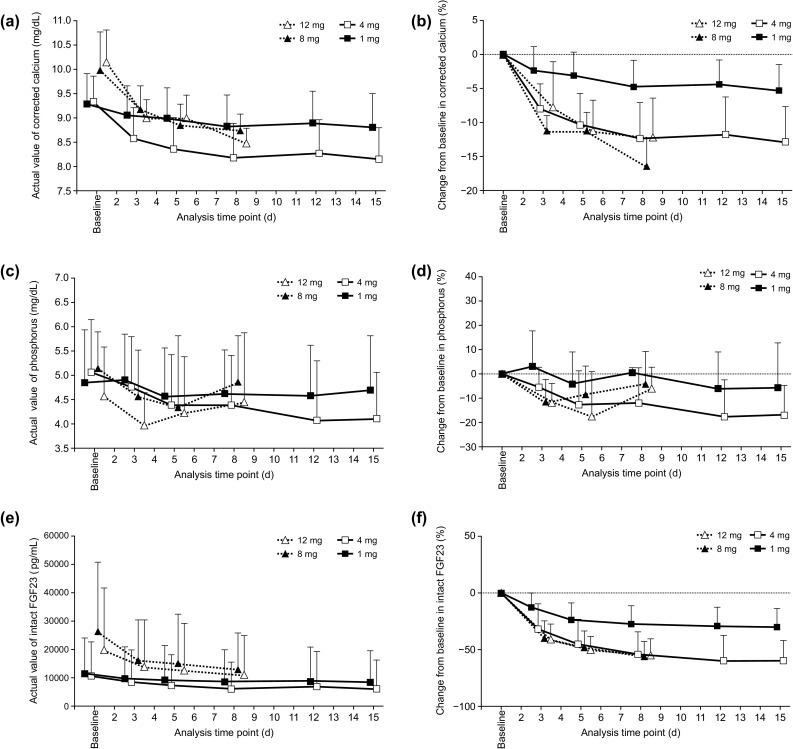
Time course of corrected calcium levels (**a, b**), serum phosphorus levels (**c, d**), and intact FGF23 levels (**e, f**) over time after multiple doses of evocalcet. *FGF23* fibroblast growth factor 23

Baseline intact PTH at the beginning of each step was similar due to the evocalcet wash out period between each step (Fig. 2b). During both the multiple-dose and extension periods, intact PTH levels remained low from day 3 to the end of the treatment following the initial decrease (Fig. 2c). In addition, although baseline serum-corrected calcium levels were different between the multiple-dose and extension periods, the serum-corrected calcium levels persisted at a lower level from day 3 until the end of the treatment (Fig. 3a, b). After administration of evocalcet, whole PTH and the concentration of ionized calcium tended to decrease during the multiple-dose and extension periods (data not shown).

The time courses of serum phosphorus and intact FGF23 during the multiple-dose and extension periods are illustrated in Fig. 3c, e, respectively. The percent change in serum phosphorus from baseline during the multiple-dose period for 1 mg and 4 mg decreased in a dose-dependent manner; however, dose dependency was not confirmed in the extension period (Fig. 3d, f). The percent change in intact FGF23 from baseline during the extension period for both 8 and 12 mg evocalcet was similar to that of 4 mg evocalcet administration during the multiple-dose period.

### Safety analysis

#### AEs

In this study, no AEs led to death, and there were no AEs related to vital signs or body weight. For the ophthalmologic examination, one patient had an AE (retinal bleeding) on day 19 of the 4 mg multiple-dose period. However, the investigator considered it to be due to diabetes, and not to be causally related to the study drug. With the exception of one patient with a moderate AE (abnormal liver function) and one patient with a severe adverse drug reaction (blood calcium decreased), all adverse events were mild in severity.

AEs occurred in 29.6% (8/27) of patients at Step 4 and 62.5% (15/24) of patients at Step 5 (Table 2a). An AE that occurred in ≥ 2 patients was nasopharyngitis in two patients (7.4%) in Step 4. In the extension period, no AEs occurred at Step 6, and 25% (1/4) of patients experienced AEs at Step 7 (Table 2b).

**Table 2:** Adverse events and drug-related adverse events during the multiple-dose period (a) and extension period (b)

(a)								
[System Organ Class] Preferred term	Adverse events				Drug-related adverse events			
	1 mg *N* = 27		4 mg *N* = 24		1 mg *N* = 27		4 mg *N* = 24	
	*n*	(%)	*n*	(%)	*n*	(%)	*n*	(%)
Patients with any events	8	29.6	15	62.5	0		9	37.5
[Cardiac disorders]	0		1	4.2	0		0	
Supraventricular tachycardia	0		1	4.2	0		0	
[Eye disorders]	0		2	8.3	0		0	
Retinal hemorrhage	0		1	4.2	0		0	
Vitreous detachment	0		1	4.2	0		0	
[Gastrointestinal disorders]	2	7.4	1	4.2	0		0	
Diarrhea	1	3.7	0		0		0	
Enterocolitis	1	3.7	0		0		0	
Nausea	0		1	4.2	0		0	
[Infections and infestations]	3	11.1	3	12.5	0		0	
Nasopharyngitis	2	7.4	3	12.5	0		0	
Pneumonia	1	3.7	0		0		0	
[Injury, poisoning and procedural complications]	0		2	8.3	0		0	
Excoriation	0		1	4.2	0		0	
Wound	0		1	4.2	0		0	
[Investigations]	2	7.4	8	33.3	0		8	33.3
Blood calcium decreased	0		8	33.3	0		8	33.3
Hemoglobin decreased	1	3.7	0		0		0	
Liver function test abnormal	1	3.7	0		0		0	
[Musculoskeletal and connective tissue disorders]	2	7.4	0		0		0	
Musculoskeletal pain	1	3.7	0		0		0	
Pain in extremity	1	3.7	0		0		0	
[Nervous system disorders]	0		1	4.2	0		1	4.2
Hypoesthesia	0		1	4.2	0		1	4.2
[Respiratory, thoracic, and mediastinal disorders]	1	3.7	0		0		0	
Oropharyngeal pain	1	3.7	0		0		0	
[Skin and subcutaneous tissue disorders]	0		1	4.2	0		0	
Hemorrhage subcutaneous	0		1	4.2	0		0	

(b)								
[System Organ Class] Preferred term	Adverse events				Drug-related adverse events			
	8 mg *N* = 5		12 mg *N* = 4		8 mg *N* = 5		12 mg *N* = 4	
	*n*	(%)	*n*	(%)	*n*	(%)	*n*	(%)
Patients with any events	0		1	25.0	0		0	
[Infections and infestations]	0		1	25.0	0		0	
Nasopharyngitis	0		1	25.0	0		0	
[Musculoskeletal and connective tissue disorders]	0		1	25.0	0		0	
Musculoskeletal pain	0		1	25.0	0		0	

#### GI-related AEs

GI-related AEs during the multiple-dose period occurred in two patients (7.4%) after multiple 1 mg evocalcet doses and included one case of diarrhea and one case of enterocolitis (Table 2a). After multiple 4 mg evocalcet doses, one patient (4.2%) experienced nausea (Table 2a). None of these AEs were causally related to evocalcet. There were no GI-related AEs or ADRs in the extension period (Table 2b).

#### Calcium-related AEs

Blood calcium decreased was defined as serum-corrected calcium levels of ≤ 7.5 mg/dL, or if the investigators judged a case to be clinically significant. Blood calcium decreased was classified as an ADR in eight patients (33.3%), and all cases occurred on either day 8 or day 12 during the multiple-dose period. In three patients (12.5%), this was classified as a significant AE that led to study discontinuation. In all three patients, the event occurred on day 8 (Step 5) of 4 mg evocalcet treatment during the multiple-dose period. Therefore, treatment with evocalcet was discontinued on day 15, upon completion of Step 5. After treatment with calcium gluconate, or during routine follow-up, the reduced blood calcium levels resolved in all three patients. In the remaining five patients, the decrease in serum-corrected calcium levels (to within the range of 7.2–7.7 mg/dL) resolved during follow-up, after finishing Step 5.

#### 12-lead ECG AEs

**Fig. 4:**
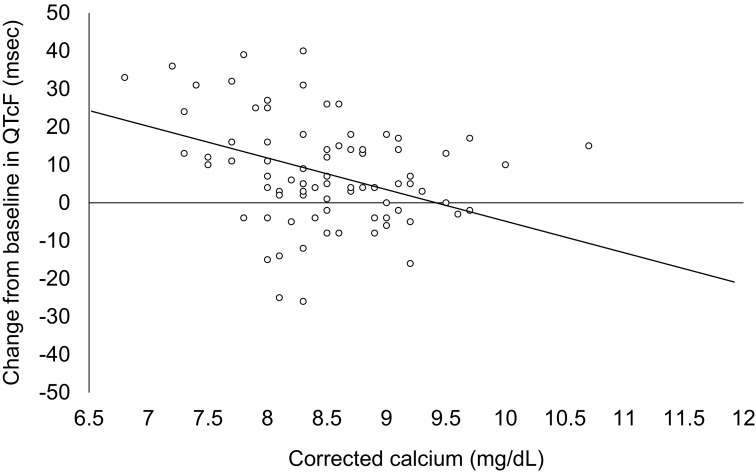
Correlation between QTcF and corrected calcium. Regression equation: change from baseline in QTcF = 78.73–8.36* corrected calcium. *QTcF* Fridericia’s corrected QT

During the multiple-dose and extension periods, there were no patients with changes from baseline exceeding 60 ms (Table 3). However, after administration of evocalcet, a negative correlation was observed between changes in the Fridericia’s corrected QT (QTcF) interval from baseline and serum-corrected calcium levels at the time ECG was assessed (Fig. 4).

**Table 3:** QTcF by category of post-dose maximum value during the multiple-dose period (a) and extension period (b)

(a)					
Parameters (unit)	Category	1 mg *N* = 27		4 mg *N* = 24	
		*n*	(%)	*n*	(%)
QTcF (msec)					
Actual value	≤ 450	18	66.7	13	54.2
	> 450 to ≤ 480	4	14.8	6	25.0
	> 480 to ≤ 500	0		1	4.2
	> 500	1	3.7	0	
Change from baseline	≤ 30	21	77.8	14	58.3
	> 30 to ≤ 60	2	7.4	4	16.7
	> 60	0		0	

(b)					
Parameters (unit)	Category	8 mg *N* = 5		12 mg *N* = 4	
		*n*	(%)	*n*	(%)
QTcF (msec)					
Actual value	≤ 450	4	80.0	3	75.0
	> 450 to ≤ 480	0		0	
	> 480 to ≤ 500	0		0	
	> 500	0		0	
Change from baseline	≤ 30	4	80.0	3	75.0
	> 30 to ≤ 60	0		0	
	> 60	0		0	

*QTcF* Fridericia’s corrected QT

## Discussion

Japanese patients receiving hemodialysis are prone to GI-related symptoms when being administered oral calcimimetics to manage SHPT. Our results show that evocalcet has the potential to improve the safety profile especially in relation to GI-related symptoms, while still providing therapeutic efficacy levels similar to cinacalcet [15, 16]. The plasma concentration of evocalcet increased in a dose-dependent manner, and evocalcet reduced intact PTH levels and calcium levels in SHPT patients receiving hemodialysis.

In contrast to cinacalcet in a pre-clinical study, evocalcet did not delay gastric emptying in rats even at a dose of 100-fold that required to achieve a significant PTH reduction [13]. In the present study, the safety profile was investigated and the data showed a low incidence of GI-related AEs even at the maximum dose of 12 mg. This agrees with our previous report in which single doses of evocalcet also produced a low incidence of GI-related AEs such as nausea and vomiting. These data, in conjunction with low discontinuation rates, suggest that adherence rates might be improved while maintaining sufficient and effective doses. Overall, evocalcet was well tolerated, although decreased blood calcium levels were reported in eight patients, resulting in treatment discontinuation in three patients (13%). These reduced blood calcium levels were resolved by treatment with calcium gluconate or routine follow-up in all three patients.

Nonetheless, evocalcet’s mechanism of action is to reduce serum calcium concentrations; therefore, this ADR is anticipated in some patients receiving calcimimetics [16, 17]. Additionally, as was the case in the single-dose study, the dosing and dose-escalation schedules as well as vitamin D and calcium formulations were fixed, and therefore, some patients would have received a dose that was higher than necessary. In fact, baseline serum-corrected calcium levels during the extension period were higher than during the multiple-dose period because only patients whose corrected calcium did not conflict with the escalation criteria could proceed to the extension period.

A correlation between QTcF prolongation and evocalcet administration was observed in this study. Calcimimetics induce blood calcium decrease, thereby prolonging the QTcF interval. This phenomenon has also been reported after cinacalcet administration [18, 19]. Therefore, careful monitoring of blood calcium levels is advised.

The single-dose study showed that the time to attain maximum plasma evocalcet concentration was approximately 4 h, and maximum plasma concentration and area under the plasma concentration–time curve increased in a dose-dependent manner [14]. After administration of multiple doses of evocalcet in the present study, the pharmacokinetic parameter *C*_trough_ showed that plasma concentrations of evocalcet were almost constant at any dose from day 3 to the end of treatment. Furthermore, evocalcet treatment at 1 and 4 mg in the multiple-dose period showed that hemodialysis is unlikely to influence evocalcet clearance rates.

Cinacalcet has a dose-dependent pharmacokinetic and pharmacodynamic profile within the range of 12.5–100 mg [15, 16]. In the single-dose evocalcet study, lower doses of evocalcet achieved a much higher plasma drug concentration than cinacalcet [14]. This may suggest that evocalcet has better bioavailability in humans as suggested in a rat model [13]. In addition, evocalcet at lower doses showed a similar suppressive effect on intact PTH as cinacalcet. Together, these data indicate that evocalcet is effective even at lower doses.

Treatment with single doses of evocalcet has previously been shown to be effective and persistent, even at doses of just 1 mg. In both the single-dose and multiple-dose studies, there were dose-dependent decreases in both intact PTH and serum-corrected calcium levels. The incidence of GI-related AEs was low in both the single-dose period and the multiple-dose period. Overall, evocalcet’s safety profile offers a potential alternative to existing calcimimetics in the management of SHPT.

## Conclusion

This study followed SHPT patients who were initially administered evocalcet in a single-dose period and then investigated the pharmacodynamics and safety profile of multiple doses of evocalcet. Therefore, although this study had a small sample size, had no placebo group, and was open label, it effectively shows that the novel oral compound evocalcet should be further tested in a larger patient cohort in the next clinical phase. Oral treatment with evocalcet suppresses intact PTH and serum-corrected calcium concentrations in a dose-dependent manner, and is a tolerable treatment option for SHPT patients receiving hemodialysis.

## Electronic supplementary material

Below is the link to the electronic supplementary material.

Supplementary material 1 (PDF 252 KB)

Supplementary material 2 (PDF 246 KB)
