# Supplementary material for: Pharmacodynamics of evocalcet for secondary hyperparathyroidism in Japanese hemodialysis patients
Source: Clin Exp Nephrol. 2018 Aug 29;23(2):258–67. doi: 10.1007/s10157-018-1635-6 (PMC6510802; doi:10.1007/s10157-018-1635-6)
Supplement: Supplementary file 1 — Supplementary material 1 (PDF 252 KB) [file 10157_2018_1635_MOESM1_ESM.pdf]

Clinical and Experimental Nephrology

Pharmacodynamics of Evocalcet for Secondary Hyperparathyroidism in Japanese Hemodialysis Patients

Takashi Shigematsu, MD, PhD, Ryutaro Shimazaki, Masafumi Fukagawa, MD, PhD and Tadao Akizawa, MD, PhD, Evocalcet Study Group

Correspondence to:

Takashi Shigematsu

Department of Nephrology, Wakayama Medical University, 811-1 Kimiidera, Wakayama city, Wakayama, 641-8509, Japan

E-mail: taki@wakayama-med.ac.jp

Supplementary Text S1

Exclusion criteria

1) Patients who had used cinacalcet hydrochloride within 2 weeks before screening.

2) Patients with changes in the dose and mode of administration of active vitamin D or its derivatives, phosphate binders, or calcium preparations within 2 weeks before screening.

3) Patients with changes in the dialysis condition (dialysate calcium level, blood purifier, prescribed duration of dialysis, prescribed frequency of dialysis) within 2 weeks before screening.

4) Patients who had received bisphosphonates, denosumab, or teriparatide preparations within 24 weeks before screening.

5) Patients who had undergone parathyroidectomy (PTx) or parathyroid intervention in the past.

6) Patients with concurrent severe heart disease (e.g., Class III or IV on the New York Heart Association (NYHA) Functional Classification).

7) Patients whose ECG waveforms were judged unsuitable for the measurement of QT interval on a 12-lead ECG by the investigator at screening.

8) Patients who had undergone surgery requiring blood transfusion, or in whom blood collection (including donation or apheresis donation) ≥200 mL had been performed within 12 weeks before screening.

9) Patients with severe hepatic function disorder (e.g., AST or ALT of ≥100 IU/L at screening).

10) Patients with poorly controlled hypertension or diabetes mellitus.

11) Pregnant, lactating, possibly pregnant women (women of childbearing potential with positive pregnancy test at screening or with negative pregnancy test but not using any contraceptive methods), or unwilling to use the adequate contraceptive method(s) according to the physician's instructions. Amenorrhea for ≥12 months after the last menstrual period without an alternative medical cause was considered as non-childbearing potential.

12) History of serious drug allergy. History of or current drug or alcohol dependence.

13) History of diagnosis and treatment of malignant tumor within 5 years before screening (excluding basal cell carcinoma or surgically resected intraepithelial carcinoma of uterine cervix).

14) Infection requiring hospitalization within 8 weeks before screening.

15) Treatment with an investigational drug in a clinical study within 12 weeks before screening.

16) Exposure to an investigational product in a prior clinical study of evocalcet.

17) Primary hyperparathyroidism.

18) Other conditions rendering the patient unfit for participation in this study at the discretion of the investigator or sub-investigator.
