# Supplementary material for: Pharmacodynamics of evocalcet for secondary hyperparathyroidism in Japanese hemodialysis patients
Source: Clin Exp Nephrol. 2018 Aug 29;23(2):258–67. doi: 10.1007/s10157-018-1635-6 (PMC6510802; doi:10.1007/s10157-018-1635-6)
Supplement: Supplementary file 2 — Supplementary material 2 (PDF 246 KB) [file 10157_2018_1635_MOESM2_ESM.pdf]

Clinical and Experimental Nephrology

Pharmacodynamics of Evocalcet for Secondary Hyperparathyroidism in Japanese Hemodialysis Patients

Takashi Shigematsu, MD, PhD, Ryutaro Shimazaki, Masafumi Fukagawa, MD, PhD and Tadao Akizawa, MD, PhD, Evocalcet Study Group

Correspondence to:

Takashi Shigematsu

Department of Nephrology, Wakayama Medical University, 811-1 Kimiidera, Wakayama city, Wakayama, 641-8509, Japan

E-mail: taki@wakayama-med.ac.jp

Supplementary Text S2

Escalation criteria

The following escalation criteria had to be met for patients to transition to the next step: no serious adverse event that could be causally related to evocalcet, a corrected serum calcium level of ≥7.5 mg/dL at each time point, and patient safety was not judged to be of concern by the investigators. If any of the escalation criteria were not met, then the following transitions were performed at each step: Step 1 (study completion); Step 2 (escalation to Step 4, without proceeding to Step 5); Step 3 (escalation to Step 4, and allowed to proceed to Step 5 but not to Step 6); and Steps 4, 5, or 6 (study completion).
